# Ultrafiltration Extract of Radix Angelica Sinensis and Radix Hedysari Attenuates Risk of Low-Dose X-Ray Radiation-Induced Myocardial Fibrosis In Vitro

**DOI:** 10.1155/2021/5580828

**Published:** 2021-04-28

**Authors:** Juan Chang, Chengxu Ma, Huan Guo, Haiqiong Ran, Guolian Chen, Yingdong Li

**Affiliations:** ^1^College of Integrated Traditional Chinese and Western Medicine, Gansu University of Chinese Medicine, Lanzhou 730000, China; ^2^The Center of Traditional Medical Diagnosis and Treatment, Gansu Provincial Hospital, Lanzhou 730000, China; ^3^Department of Endocrinology, The First Hospital of Lanzhou University, No. 1 West Donggang Road, Lanzhou, Gansu 730000, China; ^4^School of Basic Medical Sciences, Lan Zhou University, Lanzhou 730000, China

## Abstract

The risk of radiation-induced heart damage (RIHD) is a growing concern since recent advances in radiation therapy (RT) for cancer treatments have significantly improved the number of survivors. Radiation-induced myocardial fibrosis (RIMF) is the final pathological condition of RIHD and main change leading to serious cardiovascular complications following RT. The aim of this study was to investigate the effect of ultrafiltration extract of Radix Angelica Sinensis and Radix Hedysari (RAS-RH) on the proliferation, apoptosis, and reactive oxygen species (ROS) of cardiac fibroblasts after X-irradiation in vitro. The RAS-RH extract was from the Danggui Buxue decoction (DBD) in TCM. Primary cardiac fibroblasts were irradiated with 1 Gy X-ray to evaluate the effect of RAS-RH on the expression levels of cell proliferation, apoptosis, ROS, and fibrotic molecules. Our data demonstrated that X-irradiation at 1 Gy resulted in the proliferation of cardiac fibroblasts; RAS-RH attenuated the myocardial fibrosis. Furthermore, X-ray radiation reduced the apoptosis of cardiac fibroblasts; RAS-RH accelerated the apoptosis of these cells after irradiation. In addition, the damage driven by ROS in primary cardiac fibroblasts after irradiation was weakened by RAS-RH and the expression of TGF-*β*1, Col1, and *α*-SMA increased after irradiation; RAS-RH decreased the expression of these makers. Overall, these data indicate that low-dose X-ray irradiation boosts myocardial fibrosis, and the effect of RAS-RH protects against fibrosis via attenuating the proliferation and accelerating the apoptosis of myocardial fibroblasts after X-irradiation.

## 1. Introduction

Radiation therapy (RT) is an essential treatment in the management of many malignancies. However, a variety of degrees of damage can be produced after RT with an incidence as high as 20%–80% [1]. Even with precisely targeted radiation techniques and attempts to lower radiational dose with minimal secondary effects, the risk of RT complications could be still increasing [2]. Thoracic RT has been the most common application for treatment of Hodgkin's lymphoma, breast cancer, lung cancer, esophageal cancer, and other malignancies in clinic practice [3, 4]. One of the major side effects of RT is RIHD (radiation-induced heart damage) that is inevitable due to the location of heart. RIHD can include coronary artery disease, valvulitis, myocarditis, and heart failure, or even sudden cardiac death [5]. RIMF (radiation-induced myocardial fibrosis) is the end stage of RIHD and contributes to all of these conditions. There had previously found evidence of association; RIMF is mainly characterized by the proliferation of cardiac fibroblasts (CFs) and the synthesis and deposition of collagen, which are induced by a variety of cytokines and relative signaling pathways [6].

Transforming growth factor-*β*1 (TGF-*β*1) is considered a potent fibrogenic cytokine by manipulating the condition of fibrosis [7]. Regarding in vitro exposure of cardiac fibroblasts, superoxide anions stimulate the proliferation of CFs by inducing production of TGF-*β*1 [8]. ROS can influence cardiac maturation and play a pivotal role in the development of the heart [9]. It has also been reported that ROS can directly activate TGF-*β*1 and promote the proliferation of fibrosis in heart tissue. Having stated the above, ROS and TGF-*β*1 are interlinked by both feedforward and feedback mechanisms that can promote the production and deposition of collagens in the development of myocardial fibrosis [10].

There is still lack of effective treatments for RIMF, despite the existence of drugs for clinical prevention of RIMF such as Statins [11] and ACEIs [12]. In TCM (traditional Chinese medicine), Danggui Buxue decoction (DBD), a classical herbal formula, composed of Radix Angelica Sinensis and *Astragalus membranaceus* at a ratio of 1 : 5, has been used for the prevention and treatment of multiple diseases including myocardial infarction (MI) [13], anemia [14], menopausal symptoms [15], diabetic atherosclerosis [16], and non-small-cell lung cancer [17]. According to theory of TCM, RIHF is considered to be an injury of blood stasis and Qi stagnation by excess heat from radiation. The major principle of treatment is to supply Qi and nourish blood. DBD is the most typical formula for supplying both Qi and blood in TCM. Radix Hedysari is a frequently prescribed herb in clinic for benefiting Qi as Astragali Radix but has higher quantity of formononetin than Astragali Radix [18, 19]. Moreover, some studies have reported the effect of Radix Hedysari on anti-fibrosis, anti-apoptosis, and anti-oxidation in rat heart [20, 21]. Consequently, we complied with original ratio of DBD and prepared ultrafiltration extract of Radix Angelica Sinensis and Radix Hedysari instead of *Astragalus membranaceus* (RAS-RH) to supplement prophase experiments in vivo model we did [22, 23] for further research on radiation-induced heart injury.

## 2. Materials and Methods

### 2.1. Plant Materials

Ultrafiltration extract of RAS-RH from raw materials: Radix Angelica Sinensis (Umbelliferae, dried root of *Angelica sinensis* (Oliv.) Diels, 400 g), Radix Hedysari (Leguminosae, dried root of *Hedysarum polybotrys* Hand.-Mazz, 2000 g). Radix Angelica Sinensis and Radix Hedysari were got from Tasly Zhong Tian Pharmaceutical Company (Dingxi, China). Each plant material was authenticated by Ling Jin, Gansu University of Chinese Medicine (Lanzhou, China), according to the methods of the Chinese Pharmacopoeia. RAS-RH was prepared by the Gansu Academy of Membrane and Technology (Lanzhou, China); this institution has obtained the patenting rights (Patent No:CN200910021504.0, CN200910021505.5). The crude extract was separated by members of our team using chromatographic methods. The major active constituents determined including Radix Hedysari polysaccharides, Angelica ferulic acid, formononetin, astragaloside, and astragalus polysaccharides. The molecular weight cutoff (MWCO) was 100 kDa. RAS-RH was refined with water decoction at a pressure of 0.04 kPa/m^3^ at 25°C and a flow rate of 72 L/h/m^2^. Approximately, 4000 mL of filtrate was then condensed to 800 mL, which was equivalent to 1 g of RAS-RH/mL of liquid medicine [22, 24].

### 2.2. Isolation and Culture of Primary Cardiac Fibroblasts

Neonatal Wistar rats (from 1 to 3 days old) were purchased from the Research Laboratory Animal Center of Gansu University of Traditional Chinese Medicine and were prepared for Primary cardiac fibroblasts (CFs) by a modified double enzymatic dissociation of cardiac tissue [25]. The hearts were minced into small pieces around 1  mm × 1  mm × 1 mm meat paste and digested with collagenase II (Gibco, Beijing, China) in phosphate-buffered saline (PBS, pH7.4) and trypsin (0.25%, Solarbio, Beijing, China); then cells were centrifuged with PBS and collected. CFs were separated by the removal of cardiac myocytes via the selective adhesion of myocytes in a 1.5 h pre-plating interval. Cells were maintained into Dulbecco's modified Eagle Medium (DMEM) (Gibco, Beijing, China) with 10% (v/v) foetal bovine serum (FBS, Gibco, Beijing, China) and antibiotics (100u/ml penicillin and 100u/ml streptomycin, Gibco, Beijing, China) and incubated in 5% CO_2_ humidified incubator at 37°C until cells reached 70% confluence. Adherent cells were cultivated at passage 2-3 and used for further assays.

### 2.3. Groups and Treatment In Vitro

CFs were randomly divided into three groups: control group; X-ray group; RAS-RH + X-ray groups. Control group of CFs underwent sham irradiation; X-ray group was irradiated CFs once with a single 1 Gy dose of X-ray that was administered using a PRECISION lasting 38 seconds per irradiation; the exposure parameters were set to 225 KV and 13.30 mA (North Branford, Connecticut, USA); RAS-RH + X-ray groups were given RAS-RH 3, 6, 9 *μ*g/ml at 12 h, 24 h, 36 h, and 48 h after irradiation, respectively.

### 2.4. Cell Viability Assay

CFs were seeded into 96-well plates with 5 replicates for each group and pretreated in serum-free medium at for 24h. The next day, CFs was irradiated with X-ray and then incubated with RAS-RH at 12 h, 24 h, 36 h, and 48 h. According to the manufacture's indication, CFs were incubated with 10 *μ*l Cell Counting Kit-8 (CCK-8) solution (Dojindo, Kumamoto, Japan) for 2 h in 37°C and the cell viability was revealed by the Multifunctional enzyme labeling instrument, absorbance which was measured at 450 nm.

### 2.5. Apoptosis and Reactive Oxygen Species (ROS) Assay

CFs were washed three times with PBS and then were stained by annexin V-fluorescein isothiocyanate (FITC) and propidium iodide (PI) (Multisciences, Zhejiang, China); the results were evaluated by a flow cytometer (ACEA Biosciences, Hangzhou, China) with the Cellquest software (ACEA Biosciences, Hangzhou, China).

### 2.6. Total RNA Isolation and cDNA Synthesis Test by Quantitative Real-Time RT-PCR

According to indication of manufacture, extraction of Total cellular RNA by TRIZOL reagent (Ambion, Carlsbad, CA), Synthetism of First-strand cDNA was from mRNA with a Go Script™ RT reagent kit (Promega, Beijing, China). The mRNA expression levels of TGF-*β*1, Col1, *α*-SMA, and GAPDH (internal control) were analyzed by real-time PCR with a Go Taq ® qPCR Master Mix kit (Promega, Beijing, China). The data (after being normalized to GAPDH levels) were evaluated by the 2^−ΔΔ*Ct*^ method. The primers are shown in Table 1.

### 2.7. Detection of Proteins

CFs were rinsed three times with PBS. According to indication of manufacture, CFs protein was extracted by Minute^TM^ Total Protein Extraction Kit for Animal Cultured Cells and Tissues (Ca. SD-001/SN-002, Invent Biotechnologies, USA). Protein content was measured via BCA Kit (Ca. P0012, Beyotime, Shanghai, China). Samples were heated at 100°C with 5x double-color loading buffer (Ca. FD006 Hangzhou Fude Biological Technology Co, Ltd, Hangzhou, China) for 8 min to fully denature proteins. Equal amounts of protein obtained from each sample were separated on 10% SDS gels by SDS-PAGE kit (Solarbio Science & Technology Co. Ltd. Beijing, China) and transferred to 0.22um polyvinylidene difluoride (PVDF) membranes (ISEQ00010, Millipore Inc, USA). Next, membranes were blocked with 5% skim milk (Ca. 9999, CST) and then incubated overnight with primary antibodies at 4°C: 1 : 1000 GAPDH (AF7021, Affinity biosciences, OH, USA), 1 : 1000 rabbit anti-TGF-*β*1 (ab179695, Abcam, Beijing, China), 1 : 1000 rabbit anti-Col1 (ab34710, Abcam, Beijing, China), 1 : 1000 rabbit anti-*α*-SMA (AF1032, Affinity biosciences, OH, USA). Membranes were washed with TBST next day and incubated for 1h with Alexa Fluor® fluorescence-conjugated goat anti-rabbit secondary antibody (1 : 10,000 dilution, Immunoway Biotechnology, Jiangsu, China). Antibody-bound protein bands on the immunoblot were observed with chemiluminescence (Millipore, Zurich, Switzerland) by a ChemiDOC XRS + Gel imaging analysis system (BIO-RAD, USA).

### 2.8. Immunofluorescence Staining

CFs were isolated and inoculated in a 6-well plate with sterile cover glass, and the cell density was adjusted to 5 × 10^5^/m after 90% fusion. The objective area was marked with liquid blocker pen, where 50–100 *μ*l of permeabilized working solution added (G1204, Servicebio, Beijing) and then incubating for 20 minutes at room temperature. Afterward was washing three times with PBS solution, 5 minutes each. We then covered objective area with 10% donkey serum (for the case of primary antibody originated from goat) at room temperature for 30 minutes, then removed the blocking solution, and incubated cells with primary antibody (diluted with PBS appropriately) overnight at 4°C, placed in a wet box. The next day, we washed cell climbing slides three times with PBS (pH7.4), covered cell climbing slides with secondary antibody, incubated at room temperature for 50 minutes, and then incubated them with DAPI solution (G1204, Servicebio, Beijing) at room temperature for 10 minutes kept in dark place. Microscopy detection and collecting images were done by fluorescence microscopy (Panoramic DESK, P-MIDI, P250, wavelength 590 nm, Hungary).

### 2.9. Data Analysis

All of the data are presented as mean ± standard deviation (SD) unless otherwise stated. Statistical significance was determined by a one-way ANOVA, followed by the LSD or Tamhane tests. All the analysis was carried out using SPSS 17.0 (Chicago, IL) or GraphPad Prism 8. *P* < 0.05 was considered statistically significant.

## 3. Results

### 3.1. Identification of CFs and RAS-RH Inhibited the Proliferation of Cardiac Fibroblasts after X-Ray Radiation

**Figure 1:**
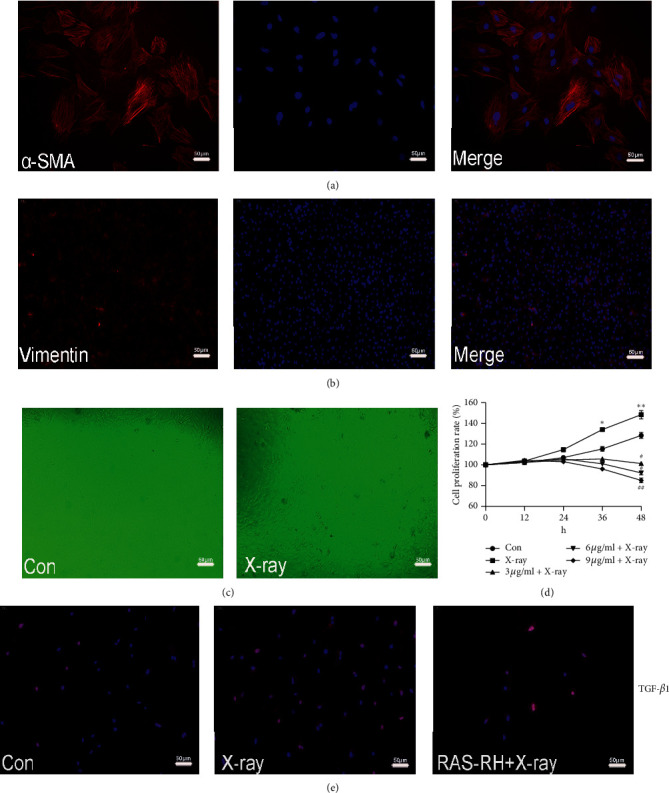
Identification of CFs and RAS-RH inhibited the proliferation of cardiac fibroblasts after X-ray radiation. (a, b) Representative images of identification of CFs by immunofluorescence staining of *α*-SMA actin and Vimentin were used to identify the purity of myocardial fibroblasts isolated by double enzyme digestion. Nuclei were stained blue; *α*-SMA and Vimentin were stained red, magnification ×200. (c) Representative results of shape of CFs by fluorescence microscope in the control group and X-ray group (at 48h after irradiation). Magnification ×40. (d) RAS-RH inhibits proliferation rate of primary CFs after X-ray radiation depended on the manner of dose-time. CFs were treated with 3, 6, and 9 *μ*g/ml of RAS-RH after X-ray irradiation for the indicated times. Proliferation rate of CFs was determined by CCK-8 assay (*n* = 5, ^*∗*^*P* < 0.05, ^*∗∗*^*P* < 0.01 vs. the control group, ^#^*P* < 0.05, ^##^*P* < 0.01 vs. X-ray group). (e) TGF-*β*1-positive cells in each group with immunofluorescence staining. Nuclei were stained blue and TGF-*β*1 stained red, magnification ×200.

So far, immunofluorescence staining of *α*-smooth muscle (*α*-SMA) actin and Vimentin as standard markers for the identification of cardiac fibroblasts still remains to be the most commonly used. More than 95% of primary CFs expressed *α*-SMA and Vimentin antigen (Figures 1(a) and 1(b)), which indicated that the method of digestion with modifications was feasible. The purity of CFs isolated by differential adhesion method was reliable, which could ensure the accuracy of follow-up test results. CFs showed irregular long spindle shape, and the cell poles were in the form of radiation. It has the characteristics of large cell volume, adherent growth, and great extensibility under the fluorescence microscope. However, morphological analysis showed there was no significant differentiation of shape in X-ray group compared to the control group at 48 h (Figure 1(c)).

The proliferation of CFs is also a factor in evaluating the occurrence of myocardial fibrosis induced by X-ray. RAS-RH on proliferation of CFs after X-ray irradiation is obvious in a time-and dose-dependent manner. As shown by CCK-8 assay (Figure 1(d)), the proliferation rate of CFs in X-ray group (148.323 ± 9.07%) was significantly higher than that in the control group (126.125 ± 2.43%) at 48 h (*P* < 0.01). However, treatment with 3, 6, 9 *μ*g/ml RAS-RH after 1 Gy X-ray irradiation, the numbers of CFs had significantly decreased; particularly, 9 *μ*g/ml RAS-RH (85.140 ± 15.24%) was significantly reduced the proliferation of CFs when compared with 3, 6 *μ*g/ml RAS-RH at 48 h (*P* < 0.01). Furthermore, 9 *μ*g/ml RAS-RH exhibited a better effect compared with 6 *μ*g/ml RAS-RH (91.333 ± 10.39%). Thus, 9 *μ*g/ml RAS-RH was used for the following studies.

As a critical factor for development of proliferation of fibrosis, the expression of TGF-*β*1 was dramatically increased in X-ray group, whereas, compared to RAS-RH + X-ray group, TGF-*β*1 was declined, shown by the immunofluorescence staining (Figure 1(e)). Compared with the control group, the fluorescence signal of the expression of TGF-*β*1 in X-ray group was significantly increased. RAS-RH + X-ray group showed a significant difference; the fluorescence signal of the expression of TGF-*β*1 was significantly decreased.

These findings suggested that X-irradiation at 1 Gy resulted in the proliferation of myocardial fibrosis in vitro that demonstrated that a low dose of X-irradiation promoted the proliferation of myocardial fibroblasts, and 9 *μ*g/ml RAS-RH partially inhibited the appearance of X-ray-induced fibrosis in vitro.

### 3.2. RAS-RH Increased the Apoptosis of CFs after X-Irradiation

**Figure 2:**
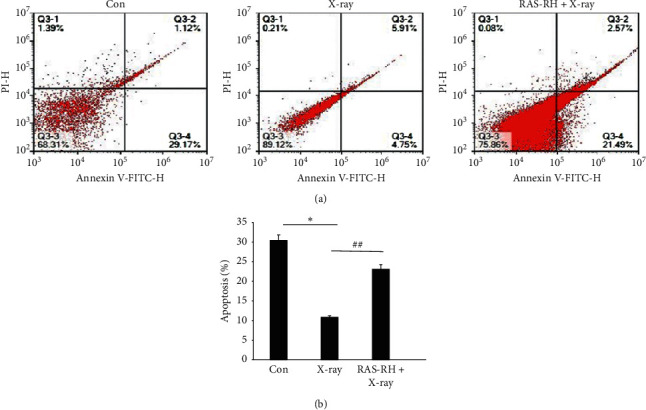
RAS-RH increased the apoptosis of CFs after X-irradiation. (a) CFs were stained with annexin V-FITC and propidium iodide. The percentages of apoptosis were detected by flow cytometry. (b) Histogram that represents a statistical analysis of image a (*n* = 3, ^*∗*^*P* < 0.05 vs. the control group, ^#^*P* < 0.05 vs. X-ray group, ^#^*P* < 0.05, ^##^*P* < 0.01 vs. X-ray group).

The above results demonstrated that 9 *μ*g/ml RAS-RH alleviated the proliferation of myocardial fibrosis after 1 Gy X-ray radiation. Therefore, we wanted to determine whether RAS-RH could induce apoptosis of CFs after X-irradiation. The percentage of apoptotic fibroblasts was detected by flow cytometry from the Con, X-ray, and RAS-RH + X-ray groups. As shown in Figures 2(a) and 2(b), we observed that, compared to the control group, the percentage of apoptotic fibroblasts was significantly decreased in the X-ray group (*P* < 0.05). On the contrary, the rate of apoptosis after treated with 9 *μ*g/ml RAS-RH increased to 23.41 ± 0.13% compared with 9.94 ± 0.60% in the X-ray group (*P* < 0.01). The results demonstrated that X-ray irradiation declined the apoptosis of CFs, whereas RAS-RH enhanced the apoptosis of CFs after irradiation, which may be via the path that RAS-RH alleviates X-irradiation-induced fibrosis.

### 3.3. Oxidative Stress from X-Irradiation Was Attenuated by RAS-RH

**Figure 3:**
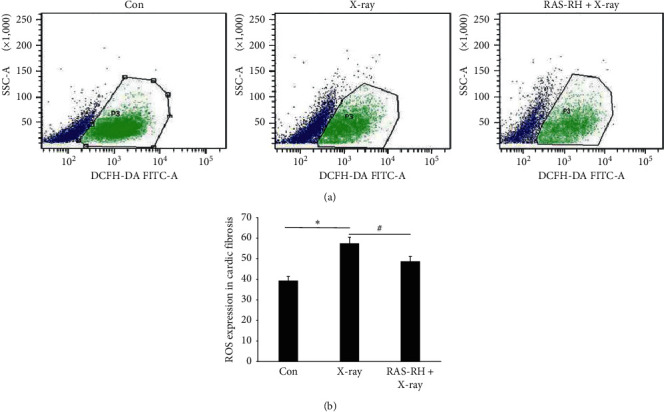
Oxidative stress from X-irradiation was attenuated by RAS-RH. ROS were analyzed by flow cytometry in the Con, X-ray, and RAS-RH + X-ray groups (*n* = 3, ^*∗*^*P* < 0.05 vs. the control group, ^#^*P* < 0.05 vs. X-ray group).

Generation of ROS brings about an acute increase in oxidative stress within cells following radiation, which has been linked to the pathogenesis of cardiac fibrosis. Therefore, we evaluated the changes in ROS formation in CFs. As shown in Figures 3(a) and 3(b), the percentage of ROS formation was significantly increased in the X-ray treatment group when compared with the control group (*P* < 0.05). Nevertheless, after treatment with RAS-RH, the percentage of ROS was dramatically reduced in the RAS-RH + X-ray group (48.6 ± 1.34%) compared with the X-ray group (57.6 ± 2.18%). The above results indicated that the damage driven by ROS in CFs after irradiation was weakened by RAS-RH.

### 3.4. RAS-RH Alleviated the Expression of TGF-*β*1, Col1, and *α*-SMA in CFs after X-Ray Radiation

Based on the above results, we further analyzed relative levels of fibrosis-related factors TGF-*β*1, Col1, and *α*-SMA, performed by western blotting detection and quantitative real-time polymerase chain reactions.

**Figure 4:**
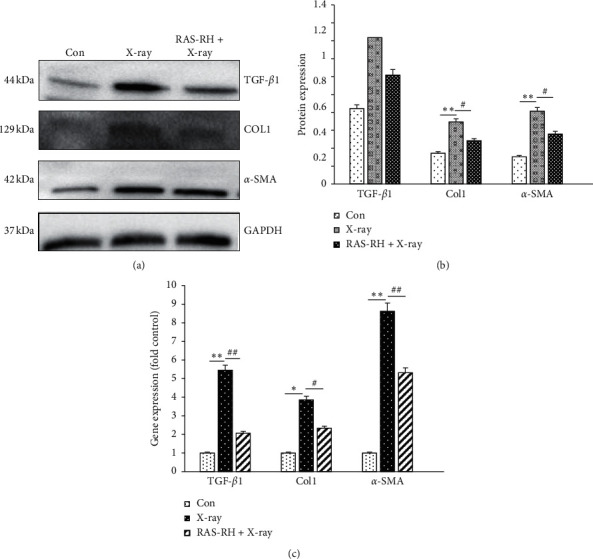
RAS-RH alleviated the expression of TGF-*β*1, Col1, and *α*-SMA in CFs after X-ray radiation. (a, b) Representative images indicate the protein level of TGF-*β*1, COL1, and *α*-SMA were analyzed with western blotting by Image-J in the Con, X-ray, and RAS-RH + X-ray groups (*n* = 3, ^*∗*^*P* < 0.05, ^*∗∗*^*P* < 0.01 vs. the control group, ^#^*P* < 0.05, ^##^*P* < 0.01 vs. X-ray group). (c) Quantitative real-time RT-PCR analysis of TGF-*β*1, COL1, and *α*-SMA mRNA levels in the Con, X-ray, and RAS-RH + X-ray groups (*n* = 3, ^*∗*^*P* < 0.05, ^*∗∗*^*P* < 0.01 vs. the control group, ^#^*P* < 0.05, ^##^*P* < 0.01 vs. X-ray group).

As vital factors for development of fibrosis, the protein expression of TGF-*β*1, Col1, and *α*-SMA was significantly increased in X-ray group as we expected when compared with that of the control group. However, we found that the protein expression level of TGF-*β*1, Col1, and *α*-SMA in RAS-RH + X-ray group was decreased as compared with X-ray group. Thus, RAS-RH has an established function in regulating fibrosis growth after X-ray radiation (Figures 4(a) and 4(b)).

To further delineate the potential mechanism involved in antifibrosis of RAS-RH, we examined the expression levels of TGF-*β*1, Col1, and *α*-SMA mRNA. The data showed increases in the level of TGF-*β*1, Col1, and *α*-SMA mRNA in the X-ray radiation group compared with that of the control group. By contrast, the level of TGF-*β*1, Col1, and *α*-SMA in the RAS-RH + X-ray group was decreased 3.388-fold, 1.532-fold, and 3.315-fold, compared with the X-ray group (Figure 4(c)).

## 4. Discussion

In past studies of RIHD, most researchers preferred to use animal models, and doses of radiation were polar opposites [26]. They wanted to confirm that the higher dose of radiation produced greater injury, but often ignoring the influence of low-dose radiation. Primary CFs were not easy to be isolated as the specific characteristics. With culture of modification, the quantity and lifespan of CFs become better than traditional methods. *α*-SMA actin and Vimentin as the standards [27] had identified the purity of CFs as showed in Figures 1(a) and 1(b). Therefore, we performed this study's in vitro model to evaluate whether low-dose radiation still causes the underlying damage for CFs. Our results revealed that 1 Gy X-ray could give rise to the proliferation and reduce the apoptosis of CFs. Thus, the results demonstrated that low-dose X-ray can establish a vitro model of RIMF and explore potential radiation injury in acute phase. Furthermore, there was related to a time-dependent manner. Generally, accumulation of radiation dosage is easy to be neglected at early stage with RT as heart is an insensitive organ on radiation. In previous studies, breast cancer patients who underwent radiotherapy even with doses <2 Gy have shown significantly increased rates of ischemic heart disease [28]. Similarly, bomb survivors in Japan and epidemiological studies on Mayak nuclear facility workers in Russia showed that doses much lower than previously assumed may increase the risk of myocardial infarction and stroke [29, 30]. Therefore, a series of subtle changes in cell biology and pathology had existed after low-dose radiation. Management of doses of radiation should be concerned, even low-dose radiation in the early stage of cancer treatment.

The TGF-*β* superfamily is composed of three isoforms: TGF-*β*1, TGF-*β*2, and TGF-*β*3. TGF-*β*1 is considered to play a critical role in the process of cell fibrosis [31]. Compared to other fibrosis factors, the expression of TGF-*β*1 mRNA is first to appear in injury. It is more obvious than the histological changes and correlates to the proportion of collagen fibers in the irradiated rat hearts [32]. TGF-*β*1 activated had been proved to promote the formation of fibrosis and associate with ROS produced under the situation of oxidative stress by irradiation. ROS is known to take part in heart vascular physiology by directly causing various forms of DNA damage in cardiomyocytes of heart tissue during radiation exposure, which can cause excess production of mitochondrial ROS leading to enhanced mitochondrial oxidative stress [33, 34]. Thus, fibroblasts exposed to radiation have increased protection against oxidative stress damage and decreased sensitivity to apoptosis which are most evidence-features in RIMF [35]. There was no remarkable difference in the shape of CFs under fluorescence microscopy between before and after X-ray irradiation at 48 h, but TGF-*β*1 in CFs examined by immunofluorescence staining showed the expression of TGF-*β*1 was increased after irradiation; on the contrary, the expression of TGF-*β*1 was reduced when intervened by RAS-RH that indicated proliferation of CFs was closely linked to activation of TGF-*β*1 after irradiation. Meanwhile, the percentage of ROS formation was upregulated in the X-ray group when compared with the control group, while the percentage of ROS was diminished in the RAS-RH + X-ray group compared with the X-ray group that implied RAS-RH could present a potential effect on anti-oxidation. From analysis of the relationship between cell number and the dosage of RAS-RH after irradiation, our experimental results showed that RAS-RH was effective in lowering the proliferation of CFs triggered by X-ray irradiation.

TGF-*β*1 is a powerful inducer of fibrosis by triggering the production of *α*-SMA and upregulation of Col1 and Col3 [36]. We determined that the protein expression of Col1 and *α*-SMA increased after X-ray radiation compared with the control group, which is not fairly obvious compared to the protein expression of TGF-*β*1 but still had significance. Furthermore, RAS-RH inhibited that the expression of TGF-*β*1, Col1, and *α*-SMA was upregulated after X-irradiation. Consistent with this finding, our data also showed obvious increases in the expression level of TGF-*β*1, Col1, and *α*-SMA mRNA after irradiation. Contrarily, the gene expression level of TGF-*β*1, Col1, and *α*-SMA was reduced after treating with 9 *μ*g/ml RAS-RH which implied that the amelioration of X-irradiation-induced cardiac fibrosis was likely related to RAS-RH.

RAS-RH had been proved that anti-fibrosis affects in vivo experiments [22]. Concurrently, we performed this study in vitro model for a further investigation on the molecular mechanism of CFs after X-ray irradiation and the effect on RAS-RH. To sum up, the results of this study showed the effect of RAS-RH on mitigating X-irradiation-induced myocardial fibrosis in vitro via attenuating the expression of TGF-*β*1, Col1, and *α*-SMA, and blocking the sustainable injury of cardiac fibrosis exposed to X-ray radiation.

Taken together, our results provide better understanding of the molecular and cellular mechanisms underlying radiation-induced toxicity in fibroblasts and show the effect of RAS-RH on RIMF. Consequently, the participation of RAS-RH seems likely to prevent X-irradiation-induced myocardial fibrosis and may be a promising substance for the treatment of RIHD.

## Figures and Tables

**Table 1:** Primer sequences used for real-time RT-PCR analyses.

Gene	Corporation	Catalog#
TGF-*β*1	FulenGen	RQP086608
*α*-SMA	FulenGen	RQP050919
Col1	FulenGen	RQP054226
GAPDH	FulenGen	RQP049537

## Data Availability

The data used to support the findings of this study are included within the article.
